# Monozygotic twins with Neurofibromatosis type 1, concordant phenotype and synchronous development of MPNST and metastasis

**DOI:** 10.1186/1471-2407-10-407

**Published:** 2010-08-05

**Authors:** German Melean, Alba Marina Hernández, María Carmen Valero, Elisabete Hernández-Imaz, Yolanda Martín, Concepción Hernández-Chico

**Affiliations:** 1 Unidad de Genética Molecular, Hospital Ramón y Cajal, Instituto Ramón y Cajal de Investigación Sanitaria (IRYCIS) and Centro de Investigación Biomédica en Red de Enfermedades Raras (CIBERER), Instituto de Salud Carlos III, Spain; 2 Beckman Institute, University of Illinois at Urbana-Champaign, USA

## Abstract

**Background:**

Neurofibromatosis type 1 is a common autosomal dominant disorder with full penetrance and variable expression. The condition predisposes individuals to the development of malignant nervous system tumours, most frequently Malignant Peripheral Nerve Sheath Tumours (MPNSTs). Previous studies indicate that genetic factors other than mutations in *NF1 *may be responsible for the condition's variable expression.

**Case report:**

Here we present data from a pair of monozygotic twins affected by Neurofibromatosis type 1 resulting from a *de novo *mutation. Both twins developed a left sciatic plexiform neurofibroma that evolved into MPNST at a similar age and they also developed pulmonary metastasis at the same age. Other concordant traits between the twins were: macrocephaly, psychomotor delay, café-au-lait spots, cutaneous neurofibromas, retroperitoneal, pleural and paraspinal neurofibromas. The main discordant features observed were tibial pseudoarthrosis, pectus carinatum, osteoporosis and thymus hyperplasia.

**Conclusions:**

This is the first report of monozygotic twins with Neurofibromatosis type 1 that develop MPNSTs, the localization and chronological evolution of which, and its metastasis, is concordant in both twins. These cases suggest that the events involved in the transformation of benign plexiform neurofibromas to MPNSTs in Neurofibromatosis type 1, follow a spatiotemporally programme that is influenced by heritable factors other than *NF1 *mutations.

## Background

Neurofibromatosis type 1 (NF1, MIM #162200) is a common autosomal dominant disorder that affects about 1 in 3,500 individuals worldwide, predisposing individuals to develop tumours in the nervous system. The condition is caused by mutations in the *NF1 *gene located at 17q11.2 that encodes neurofibromin, a 2,818 amino acid GTPase activating protein (GAP) which regulates *p21-ras *proto-oncogene activity [1]. About half of the NF1 cases represent *de novo *mutations.

NF1 is a fully penetrant condition although its presentation is variable. The main defining features are multiple "café au lait" spots (CLS), skin fold freckling, Lisch nodules and cutaneous neurofibromas [2]. Plexiform neurofibromas (PNFs) and optic gliomas are also common neoplastic manifestations associated with NF1. In NF1 patients, between 10-15% of the PNFs transform into Malignant Peripheral Nerve Sheath Tumours (MPNSTs) [3], which in turn metastasize in an early phase and are associated with a poor prognosis [4]. Other features associated with the condition are learning disabilities, macrocephaly, scoliosis, pseudoarthrosis and epilepsy.

Although many different mutations have been reported in NF1 [5], no genotype-phenotype relationships have been found, except for large deletions encompassing the entire *NF1 *gene and its flanking chromosomal regions (*NF1 *microdeletions), or a 3 bp deletion in exon 17 of *NF1 *(c.2970-2972delAAT; p.990delM). *NF1 *microdeletions are associated with the appearance of a large number of cutaneous neurofibromas, dysmorphic features, learning disabilities and an elevated risk of MPNSTs [6,7]. By contrast, the small deletion in exon 17 is associated with a milder phenotype and the absence of neurofibromas [8].

A contribution of modifying genes not linked to the *NF1 *locus might explain the variable expression of this condition. This hypothesis is supported by phenotype correlation studies in different groups of affected relatives, including monozygotic (MZ) twins [9,10], and through the analysis of intrafamilial associations between the clinical features of NF1 [11,12].

Better understanding of MPNST variable occurrence in NF1 has been elusive, due to the tumor's low frequency and the uncommon presentation in multiple members of the same family. Here we report an illustrative case of NF1 MZ twins with PNF in similar anatomical location that transformed to MPNST and produced pulmonary metastasis, simultaneously in both twins.

## Case Report

Two 27 year-old male MZ twins were referred to our Unit for molecular analysis of the *NF1 *gene. The probands were born after an uncomplicated pregnancy to healthy non-consanguineous unaffected parents, with no prior family history of NF1. The twins have an unaffected brother 4-years older who suffered mild psychomotor delay and dysphasia in early childhood. The twins grew to adulthood and they are currently still living together.

Both twins presented with minimal cutaneous manifestations, including approximately 15 CLS and a few cutaneous neurofibromas (detailed clinical data is reported in Table 1). A relevant tumour phenotype was observed in both twins. In "Twin 1", MRI performed at 20 years of age detected one PNF of the left sciatic popliteal nerve and many small neurofibromas in both legs surrounding the femoropopliteous vessels. Although the PNF was resected due to the distal pain in the leg, at 22 years of age a further surgical intervention was performed to resect a tumour detected by MRI in the same region. Pathological analysis of the resected tissue diagnosed a MPNST of the sciatic popliteal nerve and a PNF. Although the patient was subjected to a cycle of radiotherapy after surgery, the MPNST recurred twice 2 and 4 years later. These tumours were treated surgically, leading to the amputation of the left leg in the last operation. In "Twin 2", MRI examination at 20 years of age identified bilateral PNFs of the sciatic nerves and at 24 years of age, the tumour in the left leg was surgically resected due to its rapid growth and malignant radiological aspect. The pathological analysis of the resected tissue diagnosed high grade MPNST of the left sciatic nerve and hence, a cycle of radiotherapy was administered after surgery.

**Table 1:** Clinical features of "Twin 1" and "Twin 2".

Age at diagnosis	FEATURE	TWIN 1	TWIN 2
At birth	CLS	+	+
	Macrocephaly	+	+
	Pectus carinatum	+	-
	Scoliosis	+	-
	Tibial pseudoarthrosis	+	-

Childhood	Mild psychomotor delay	+	+
	Developmental dysphasia	+	+
	Hyperkinesia/attention disorder	+	+
	Hypotonia	-	+
	Strabismus	-	+
	Dental development delay	+	-
	Right valgus foot	+	+
	Bilateral flat feet	+	+
	Normal cranial CT	+	+
	Normal ophthalmologic examination	+	+

14 Y/O	Radial intraneural neurofibroma	+	-

20 Y/O	Plexiform neurofibroma of the left sciatic nerve	+	+ (bilateral)
	Multiple neurofibromas surrounding femoropopliteous vessels	+	-

22 - 24 Y/O	MPNST of the left sciatic nerve	+ (22 Y/O)	+ (24 Y/O)

24-27 Y/O	MPNST recurrences	+	-
	Osteoporosis	-	+
	Thymus hyperplasia	-	+
	Bilateral gynecomastia	-	+
	Left lung focal pleural thickening	+	+
	Pleural neurofibroma	+ (multiple)	+ (one)
	Hepatic hypodense lesion	+	+
	Paraspinal plexiform neurofibroma	-	+
	Psoas plexiform neurofibroma	+	-
	Multiple retroperitoneal neurofibromas	+	+

27 Y/O	Pulmonary MPNST metastasis	+ (right lung)	+ (left lung)

A pulmonary metastatic MPNST was detected in both twins at 27 years of age. "Twin 1" developed the metastasis in the lower lobule of the right lung, while "Twin 2" presented the metastasis in the upper lobe of the left lung. The concordant and discordant manifestations associated with NF1 in these twins are presented in Table 1.

### Mutational *NF1 *analysis and twins' zygosity test

Lymphocyte RNA was obtained from fresh peripheral blood and RT-PCR was performed to amplify overlapping cDNA fragments that covered the entire gene. The PCR products were analyzed by Denaturing High Performance Liquid Chromatography (DHPLC) and the positive fragments in the DHPLC analysis were sequenced. The mutations found were confirmed by direct sequencing of the genomic DNA and in this way, the c.4537 C→T mutation (Arg1513X) was found in exon 27a of the *NF1 *gene from both twins. The mutation was not present in the twins' parents or brother.

Twins' zygosity was confirmed by analysing 14 polymorphic microsatellite markers at different chromosomal loci. Informed consent for genetic analysis was obtained from all family members included in this study.

## Case Discussion

We report here a pair of NF1 MZ twins with remarkable concordance in their tumour phenotype. The c.4537 C→T disease-causing mutation was found in both twins and it is a nonsense mutation that has been recurrently found in subjects with NF1 [13], although it does not appear to be associated with any particular clinical feature of the disease.

Macrocephaly, CLS and cutaneous neurofibromas are concordant traits in our twins, as was reported previously in a cohort of six MZ twin pairs [9]. Concordance among more distant relatives was weaker, indicating that genetic factors other than the mutant *NF1 *allele influence the expression of these traits [9-11]. Psychomotor delay and learning disabilities develop in 30-60% of individuals affected by NF1, and learning disabilities were seen to be 100% concordant in NF1 MZ twins [9,10]. In this sense, our probands presented similar alterations, although both responded well to therapy. However, it is not possible to confirm that this feature is associated with NF1 as the twins' older unaffected brother also displayed learning problems during early childhood and responded well to therapy.

Scoliosis and tibial dysplasia were only evident in "Twin 1", consistent with the low correlation coefficient of 0.17 established in 6 MZ twins [10], even though scoliosis was previously reported to be a concordant feature in another 3/3 NF1 MZ twins [9]. There is no prior information on tibial dysplasia in MZ twins with NF1 and the osteoporosis developed by "Twin 2" is probably associated with parathyroid gland disease [14].

PNFs were previously found to have a strong concordance among NF1 MZ twins, yet while concordance was lower in more distant relatives, loci other than *NF1 *were thought to modulate this feature [9,10]. Our observations are in accordance with such an influence.

To our knowledge, this is the first report of NF1 MZ twins with MPNSTs and what is more, both twins developed MPNSTs at similar ages and at the same sciatic PNF location. Indeed, pulmonary metastases also appeared simultaneously in both twins at 27 years of age. Significantly, "Twin 2" developed another PNF in the right sciatic nerve that is still under surveillance.

Our current understanding of the molecular mechanisms underlying PNF transformation into a MPNST is still limited. Two events seem to be essential for the development of NF1 tumours: the inactivation of the remaining wild type *NF1 *allele (somatic mutation) in a schwann cell precursor; and a tumour microenvironment produced by the *NF1 *haploinsufficency in other tumoural elements (fibroblasts, perineural-like fibroblasts and mast cells). This microenvironment provides crucial mitogenic signals to homozygous mutated *NF1 *schwann cells [15]. In addition to the double mutation of the *NF1 *gene, additional genetic alterations also seem to be required to permit the transformation of benign PNF into MPNST. These alterations include inactivation of cell-cycle associated genes such as *TP53*, *RB1 *and *CDKN2A* which encodes p16(*INK4A*) and p14(*ARF*) [16,17]. However, a recent study reported LOH of *TP53 *and *RB1 *in a minority of cutaneous neurofibromas (4.5 and 1.1% respectively) [16]. Gene expression profiling in NF1-associated benign and malignant tumours identified relatively widespread deregulation of genes in developing schawnn cells and in particular, the *SOX9 *gene seems to be essential for MPNST cell survival [18]. Microsatellite instability (MSI) has also been associated with peripheral nerve NF1 tumours, both benign and malignant [16,19] Malignant tumours present higher levels of MSI than benign ones and these findings suggest that mismatch repair genes (MMR) may also participate in the malignant transformation of NF1-associated tumours [20,21]. Unfortunately, we could not perform further molecular investigations in our twins since no further tumour tissue was available.

The most exceptional feature in our twins is that concordance was not only detected in terms of the presence of a MPNST in both twins but also, in the location and chronological evolution. An interesting theory has been proposed that may be related with this concordance, based on the assumption that the potential for angiogenesis varies spatiotemporally in different vascular fields. Hence, it was suggested that *NF1 *deficiency may disrupt some vascular fields, producing anatomical regions that are prone to develop tumours and malformations [22].

Previous reports in MZ twins with *BRCA1 *and *BRCA2 *mutations suggest that an analogous regulated program may also occur in other kinds of hereditary tumours. Similar to this case, in MZ twins with mutations in *BRCA1 *and *BRCA2 *tumours were detected at the same approximate location and same age [23,24].

## Conclusions

For the first time, we report NF1 MZ twins concordant for MPNSTs, with a similar clinical presentation of sciatic nerve PNF, as well as a similar malignant and metastatic evolution. This case indicates that the events needed for the transformation of a PNF to MPNST, and their subsequent metastasis, follow a chronological and spatially regulated programme, where heritable factors other than the *NF1 *mutation exert a strong influence.

## Competing interests

The authors declare that they have no competing interests.

## Authors' contributions

**GM **and **AMH **were involved in the patient management and writing of the article; **MCV**, **EHI **and **YM **in the molecular analysis and critical revision of the manuscript; and **CHC **was responsible for overseeing the study, and for the writing and the critical revision of the article. All authors read and approved this manuscript.

## Pre-publication history

The pre-publication history for this paper can be accessed here:

http://www.biomedcentral.com/1471-2407/10/407/prepub
